# Bullying victimization and mental health before and during the COVID-19 pandemic

**DOI:** 10.3389/frcha.2024.1411265

**Published:** 2024-05-22

**Authors:** Ann H. Farrell, Heather Brittain, Amanda Krygsman, Tracy Vaillancourt

**Affiliations:** ^1^ Department of Child and Youth Studies, Brock University, St. Catharines, ON, Canada; ^2^ Counselling Psychology, Faculty of Education, University of Ottawa, Ottawa, ON, Canada; ^3^ Faculty of Social Sciences, School of Psychology, University of Ottawa, Ottawa, ON, Canada

**Keywords:** bullying victimization, mental health, COVID-19, students, population-based

## Abstract

**Introduction:**

Bullying victimization is associated with numerous mental health difficulties yet studies from early in the COVID-19 pandemic revealed significant decreases in bullying victimization but significant increases in mental health difficulties for many children and adolescents. It is unclear whether the decrease in bullying victimization early in the pandemic translated to weaker associations between bullying victimization and mental health difficulties.

**Methods:**

Using a population-based design, we examined whether the correlations between bullying victimization and mental health difficulties were significantly weaker in magnitude during the COVID-19 pandemic compared to before the pandemic in a sample of 6,578 Canadian students in grades 4–12. Students were randomly assigned to report on their bullying and mental health experiences either during the school year before the pandemic or the school year during the pandemic. Only students who reported experiences of victimization were included in the present study as questions on mental health were specifically on difficulties experienced due to victimization.

**Results:**

As expected, overall bullying victimization and mental health difficulties were significantly correlated before and during the pandemic, but correlations were significantly weaker in magnitude during the pandemic for girls and secondary students. Significant decreases in correlation magnitude were also found predominately for general, verbal, and social forms of bullying victimization, but not for physical and cyber victimization. Among students who reported victimization, we also found significantly lower means for mental health difficulties and most forms of bullying victimization during the pandemic compared to pre-pandemic.

**Discussion:**

Findings indicate a strong coupling of bullying victimization and mental health difficulties, particularly before the pandemic, and the need to reduce these associations to improve the well-being of children and adolescents.

## 1 Introduction

Numerous studies show a strong association between bullying victimization and mental health difficulties [e.g., see meta-analysis by Moore et al. (1)]. Bullying is considered an intentionally harmful behavior conducted within the context of a power imbalance that can be repetitive and used to obtain resources such as social status and power (2). Across the world, 30% of children and adolescents experience victimization (3). In addition, 10% of children and adolescents experience victimization frequently (4). Given the intentional harm aimed at a target, it is not surprising that bullying victimization is associated with psychological difficulties including depression, anxiety, and suicidality [see McDougall & Vaillancourt (5) for a review]. Longitudinal studies have revealed causal pathways with childhood bullying victimization predicting depression, anxiety, and suicidality symptoms 30–40 years later [i.e., interpersonal risk pathway (6)] and depression symptoms predicting bullying victimization across 8 years of childhood and adolescence [i.e., symptoms driven pathway (7)]. Meta-analytic findings also support the strong coupling of bullying victimization and mental health difficulties such as depression (1). Meta-analyses also support gender differences, with boys consistently involved in bullying perpetration and victimization more frequently than girls (8), although Canadian studies have shown that girls are victimized more than boys, and boys perpetrate more than girls (9, 10). In addition, bullying prevalence rates have been shown to peak during early adolescence [e.g., (11)].

Belonging to secure and stable social groups is a fundamental motivator of human survival (12). Being targeted by the peer group interrupts this critical need to belong, adversely impacting an individual's perception of themselves and others. Preventing bullying victimization is therefore critical for preventing mental health difficulties in children and adolescents.

Evidence from early in the COVID-19 pandemic indicated a significant decrease in overall bullying prevalence rates likely due to pandemic mitigation practices which decreased peer contact and increased student supervision [see Vaillancourt et al. (13) for a review]. This decrease in bullying victimization was not however commensurate with decreases in mental health difficulties. Indeed, when examining mental health difficulties alone, meta-analytic findings showed significant increases [e.g., increased depression, anxiety, suicidality, eating disorders (14–16)]. These increases have been attributed to pre-pandemic mental health difficulties combined with social isolation during the pandemic (17). In the present study, we explored whether the association between bullying victimization and mental health difficulties among children and adolescents was significantly different before the COVID-19 pandemic compared to the first year of the pandemic.

### 1.1 Bullying before and during the COVID-19 pandemic

The COVID-19 pandemic resulted in drastic changes to daily routine to prevent the spread of SARS-CoV-2 including in-person school closures and cancellations of in-person activities. Evidence from early in the pandemic showed significantly lower levels of bullying involvement relative to known prevalence rates from before the pandemic. For example, in some of the first available data, UNICEF (18) reported that 17% of individuals ages 13–24 reported being bullied less during the pandemic when compared to before the pandemic. In a sample of Chinese adolescents ages 14–17, bullying was measured before the pandemic and at three time points during the pandemic: February 2020 (lockdown), April 2020 (restrictions eased), and June 2020 [schools reopened (19)]. In this study, victimization rates were lower during the pandemic lockdown compared to before the pandemic. In another study from early in the pandemic, students across grades 4–12 were randomized into answering about bullying either before the pandemic or during the pandemic (20). The pandemic group reported on bullying since the Fall of 2020, once in-person schooling was reopened. There were significantly lower rates of bullying perpetration and victimization. For overall bullying victimization, rates were 59.8% before the pandemic and 39.5% during the pandemic, and for overall bullying perpetration, rates were 24.7% before the pandemic and 13.0% during the pandemic. Also, in the study by Vaillancourt et al. (20), when examining specific forms of bullying, decreases were found for perpetration and victimization for all forms including verbal, physical, relational, and cyber. These declines were attributed to pandemic mitigation practices during in-person school (e.g., social distancing, increased teacher supervision), which could have unintentionally impacted students' peer interactions including bullying. Similarly, Repo et al. (21) found significant decreases in bullying victimization in Finnish students ages 10–16 when comparing prevalence rates during online schooling in the Spring of 2020 to prevalence rates from two cohorts of students from 2019 and 2017.

Although much of the evidence from early in the pandemic during the initial lockdowns and school re-openings showed decreases in bullying prevalence rates, evidence from other studies conducted after the first year of the pandemic when schools were in-person indicate increases [see Vaillancourt et al. (13) for a detailed review]. In two studies, one conducted in China (22) and one conducted in Norway (23), bullying prevalence rates were higher in 2021 (when schools were in-person) compared to different cohorts of students from before the pandemic. This increase was attributed by the researchers to stressors at school during the pandemic, including teacher responsibilities (e.g., enforcing social distancing), which could have reduced bullying prevention practices (23). Some researchers also found that cyberbullying prevalence rates increased. Patchin and Hinduja (24) found that the prevalence of being cyberbullied increased from 17.2% in 2019 to 23.2% in 2021. However, some recent systematic reviews and meta-analyses found mixed results. Huang et al. (25) found that cyberbullying perpetration decreased during the pandemic while cyberbullying victimization reflected no changes, whereas Sorrentino et al. (26) found cyberbullying perpetration and victimization decreased in some countries, but increased in others. Thus, it is possible that in some regions where students experienced longer durations of pandemic restrictions, online socialization may have contributed to more opportunities for cyberbullying. More recently, Patte et al. (27) found that the odds of bullying victimization decreased at the start of the pandemic (2020/2021 academic year) in cross-sectional and longitudinal samples. However, in the following academic year (2021/2022) these odds increased to surpass pre-pandemic prevalence rates. The wide range of variability in study design (cross-sectional, longitudinal), measures of bullying (e.g., reference period), and length and type of school-based pandemic related restrictions are likely to have contributed to these mixed results [see (13)]. These findings suggest that bullying prevalence rates generally declined early in the pandemic even when schools re-opened in 2020 (20) and increased after the first year of the pandemic [e.g., (23)].

### 1.2 Child and adolescent mental health before and during the COVID-19 pandemic

In contrast to bullying rates which decreased then increased, evidence on child and adolescent mental health during the pandemic demonstrated a more consistent pattern. Before the pandemic, mental health difficulties were already prevalent among children and adolescents. Data from the Global Burden of Disease Study indicated that 1 in 10 children and adolescents between ages 5 and 24 had at least one mental disorder with a prevalence rate of 11.63% (28). Meta-analyses revealed a continued high prevalence of mental health difficulties after the onset of the COVID-19 pandemic. Racine et al. (29) examined studies from early in the pandemic conducted until March 2021 and found that the prevalence rates of clinically elevated depression and anxiety symptoms had doubled, with depression symptoms at 25.2% and anxiety symptoms at 20.5%. Alizadeh et al. (30) found similar prevalence rates as Racine et al. In their meta-analysis of cross-sectional and longitudinal studies conducted until December 2022, Alizadeh et al. found the prevalence rates of depression symptoms was 23% and anxiety symptoms was 26%.

In a recent meta-analysis of longitudinal studies published between January 2020 and May 2022 that included pre-pandemic measurement, Madigan et al. (15) found significant increases in depression and anxiety symptoms from before to during the pandemic among children and adolescents, with the effect of depression seen particularly among girls. In addition, two meta-analyses found significant increases in emergency department visits during the pandemic compared to before the pandemic among children and adolescents for attempted suicide and suicide ideation (14) and eating disorders (16). For eating disorders and suicide attempts, the effect was stronger for girls compared to boys.

### 1.3 Bullying and mental health before and during the COVID-19 pandemic

Considering these changes in bullying and mental health, it is unclear whether the associations between bullying victimization and mental health difficulties were also impacted by the pandemic. Much of the research during the pandemic has examined bullying and mental health independently. The limited number of studies examining both bullying and mental health provide some insight on their associations and effect sizes during the pandemic. For example, Repo et al. (21) found associations between bullying victimization and indicators of mental health (anxiety, loneliness) before and during the pandemic. The correlations were all significant and looked smaller during the pandemic than before the pandemic (*r*s = .15 during pandemic; *r* = .21–.26 before pandemic), but these correlations were not compared statistically. Instead, the focus was on comparing bullied students to non-bullied students.

In another study, Garthe et al. (31) examined cyber victimization and mental health outcomes in students with a mean age of 11.75 in the fall of 2019 and spring of 2021 during remote schooling. Like Repo et al., Garthe et al. provided correlations between cyber victimization and indicators of mental health but did not compare their correlations statistically. The correlations were all significant and again appeared to be weaker during the pandemic (*r* = .09–.15) compared to before the pandemic (*r* = .21–.30). The researchers also found that cyber victimization decreased whereas mental health difficulties increased, but higher levels of pre-pandemic cyber victimization predicted increases in depression, anxiety, and social stress symptoms during the pandemic. Finally, Basilici et al. (32) examined emotional symptoms and bullying victimization in Italian adolescents ages 12–19 across three timepoints (pre-pandemic January/February 2020, February/March 2021, May/June 2021). Again, the correlations during the latter two pandemic time points appeared smaller than before the pandemic (*r* = .22 and .28 vs. .32), but they were not compared statistically. The researchers also found that bullying victimization decreased and emotional problems increased, but individuals starting with lower pre-pandemic victimization increased more in emotional problems.

### 1.4 Current study aims

Since bullying prevalence rates were found to decrease during the pandemic (20), while mental health difficulties were found to increase during this period (15), our study aim was to examine whether the strength of association between bullying victimization and mental health difficulties was significantly different from before the COVID-19 pandemic compared to after the pandemic onset using a population-based randomized design in a sample of Canadian students in Grades 4–12. Given the focus of existing studies has been on overall mental health, we specifically examined mental health difficulties due to victimization. This way, we could examine (1) the association between bullying victimization and mental health difficulties specifically due to victimization, and (2) changes in mental health specifically due to victimization before compared to during the pandemic.

We predicted that higher levels of bullying victimization would be significantly associated with higher levels of mental health difficulties attributed to victimization both before and during the pandemic. However, given that bullying prevalence rates significantly decreased during the pandemic, we predicted that the magnitude of the association between bullying victimization and mental health attributed to victimization would be weaker during the pandemic compared to before the pandemic. We also predicted that mean levels of bullying victimization and mental health due to victimization would be significantly lower during the pandemic compared to before the pandemic. We examined these patterns by gender (girls, boys), age (elementary, secondary), and form (general, physical, verbal, social, cyber bullying victimization). Previous population-based Canadian studies have shown that girls are often targeted by bullying more often than boys, bullying is often higher among elementary students that secondary school students, and verbal and social forms of bullying are more prevalent than physical (9, 10). We expected the same pattern across gender, age, and form.

## 2 Materials and methods

### 2.1 Participants

Participants included 6,578 students in Grades 4–12 from a larger study from 2020 as part of a Safe Schools audit examining bullying and school safety for a large school district in Ontario, Canada. Schools were randomized into one of two conditions. In the “pre-COVID” condition, students responded to questionnaires based on their experiences during the previous school year before the COVID-19 pandemic onset (September 2019–March 2020). In the current “pandemic condition”, students responded to questionnaires based on their experiences during the school year since the COVID-19 pandemic onset (September 2020–November 2020). See Vaillancourt et al. (20) for full details on participants and study design. To be included in the analytic sample in the present study, participants had to report being bullied and have responses on mental health. Only students reporting being bullied were asked follow-up questions on mental health experiences specifically related to their experiences with bullying victimization. Thus, participants who did not report any victimization experiences were excluded. This criterion resulted in an analytic sample of 3,291 students (*M*_age_ = 12.63, *SD* = 2.31, *Min* = 8, *Max* = 19; 51% girls, *n* = 1,678; 42.4% boys, *n* = 1,394; 3.1% gender diverse^1^, *n* = 101, 3.6% missing, *n* = 118). In the pre-COVID condition, there were 2,262 students (68.7% of analytic sample). In the pandemic condition, there were 1,029 students (31.3% of analytic sample). Sample sizes for the two conditions are not equal as randomization was at the school level and student populations at each school varied. See Table 1 for gender and race by condition (pre-COVID, pandemic).

**Table 1:** Frequencies and percentages of gender and race by Pre-COVID and pandemic conditions.

	Pre-Covid *n* (%)	Pandemic *n* (%)
Gender		
Girls	1,193 (52.7%)	485 (47.1%)
Boys	923 (40.8%)	471 (45.8%)
Gender diverse	62 (2.7%)	39 (3.8%)
Missing	84 (3.7%)	34 (3.3%)
Race		
White	984 (43.5%)	459 (44.6%)
Underrepresented race	614 (27.1%)	321 (31.2%)
Missing	664 (29.4%)	249 (24.2%)
*n*	2,262	1,029

### 2.2 Procedure

Student participants required both passive parental consent and student assent/consent to participate. Most parents of students (94.0%) provided consent for their child to participate (remaining 6.0% opted out) and most students provided assent/consent to participate (97.3%). In the fall of 2020, schools in Ontario had three possible learning modes because of the pandemic: (1) full-time in-person learning for Grades 4–8, (2) full-time virtual e-learning for Grades 4–12, and (3) blended in-person/on-line learning for secondary students in Grades 9–12. Therefore, for students attending blended or full-time in-person, classroom teachers provided school electronic devices for students to access and complete the study survey. For students in full-time virtual e-learning, parents of students were provided with instructions for completing the survey at home. Participants were told that they could skip any question and that all responses were anonymous as no identifying information was collected. Technical support was also available for any parents, students, or teachers. All measures were previously validated in over 25,000 students (9, 10).

### 2.3 Measures

#### 2.3.1 Bullying victimization

Participants were first provided with a definition of bullying to ensure the difference between bullying and general aggression. For students in-person, teachers read the definition out loud before students started the survey. For students online, the definition was provided with instructions. Bullying victimization was assessed with five self-report items adapted from the Olweus Bully/Victim Questionnaire (2, 9, 10). Participants were first asked, “How often were you bullied by another student(s)?” to assess general bullying victimization, followed by questions specifically on physical, verbal, social, and cyber bullying victimization. Items were rated on a five-point scale (1 = *not at all* to 5 = *many times a week*) and averaged to create a composite score, with higher scores indicating higher levels of victimization. Only participants with a score higher than 1 (i.e., experienced bullying victimization) were included in the present study and presented with subsequent questions on mental health related to bullying victimization. Cronbach's alpha for the analytic sample was acceptable for both conditions (pandemic *α* = .69; pre-COVID *α* = .77).

#### 2.3.2 Mental health

For participants who reported being targeted by bullying, 13 follow-up questions on mental health specifically related to being victimized were presented (9, 10). Participants responded to “Because I was bullied…” with each item reflecting a different area of mental health impacted. Sample items include, “…I have been sad.”, “I have felt sick (e.g., headaches, stomach aches, trouble sleeping).”, “I have felt powerless.”, and “I have felt hopeless.”. Items were rated on a five-point scale (1 = *not at all* to 5 = *many times a week*) and averaged to create a composite score, with higher scores indicating higher levels of mental health difficulties. Cronbach's alpha for the analytic sample was excellent for both conditions (pandemic *α* = .93; pre-COVID *α* = .92).

### 2.4 Analytic plan

Among students experiencing bullying victimization, we examined whether the correlation between bullying victimization and mental health difficulties during the COVID-19 pandemic was significantly different in strength from the correlation between bullying victimization and mental health difficulties before the COVID-19 pandemic. The primary analyses involved running bivariate correlations between bullying victimization and mental health difficulties by condition (pre-COVID, pandemic) and conducting a Fisher's *r*-to-*z* test to determine if the correlations were significantly different in strength from one another. Given the known differences in bullying and mental health by gender, age, and form of bullying, we examined these correlations for: (1) overall bullying victimization and mental health for (1a) the overall sample, (1b) by gender (boys, girls), and (1c) by grade (elementary, secondary). These correlations were then repeated by form of bullying victimization including: (2) general, (3) physical, (4) verbal, (5) social, and (6) cyber. SPSS version 29 was used for all analyses. We also ran independent *t*-tests examining mean differences between pre-COVID and pandemic conditions on mental health and bullying victimization (overall), general, physical, verbal, social, and cyber. Finally, we applied the Benjamini-Hochberg (BH) correction for multiple testing for all statistical tests to reduce Type I error (33).

## 3 Results

First, confirmatory factor analysis with principal axis factoring was conducted to examine the psychometric properties of the mental health variable within each of the two conditions. One factor accounted for 47.62% in the pre-COVID condition and 49.25% in the pandemic condition, indicating that a one factor model was appropriate for the mental health items. Factor loadings were also high, ranging from .48 to .82 for the pre-COVID condition and .49 to .87 for the pandemic condition.

Next, the primary analyses were conducted, which involved the correlations between bullying victimization and mental health. See Tables 2–5 for the means, standard deviations, and bivariate correlations for bullying victimization and mental health by condition. All correlations between bullying victimization and mental health were significant and positive, regardless of the condition (pre-COVID, pandemic) and form (i.e., overall, Table 2; general, physical, verbal, social, and cyber, Tables 3–5). In addition, correlations were significant and positive within each gender (boys, girls; Table 4) and grade (elementary, secondary; Table 5) regardless of the condition and form. The effect sizes for correlations ranged from small-to-moderate to moderate-to-large for both pre-COVID (*r* = .31–.75) and pandemic (*r* = .28–.67) conditions.

**Table 2:** Means, standard deviations, and bivariate correlations between overall bullying victimization and mental health before and during the COVID-19 pandemic.

	Pre-Covid *n*	Pre-Covid *M(SD)*	Pandemic *n*	Pandemic *M(SD)*	Pre-Covid *r(n)*	Pandemic *r(n)*	*r*-to-*z* test
Overall							
Victimization	2,312	1.80 (0.73)	1,052	1.67 (0.63)	.706, *p* < .001 (2,262)	.665, *p* < .001 (1,029)	***z* = 2.06,** ***p* = .039**
Mental health	2,262	1.61 (0.74)	1,029	1.51 (0.71)			
Gender							
Girls							
Victimization	1,225	1.79 (0.73)	496	1.65 (0.59)	.708, *p* < .001 (1,193)	.642, *p* < .001 (485)	***z* = 2.25, *p* = .024**
Mental health	1,193	1.65 (0.76)	485	1.54 (0.66)			
*Boys*							
Victimization	938	1.78 (0.71)	480	1.64 (0.64)	.690, *p* < .001 (923)	.667, *p* < .001 (471)	*z* = 0.75, *p* = .453
Mental health	923	1.50 (0.65)	471	1.40 (0.65)			
Grade							
Elementary							
Victimization	1,541	1.83 (0.73)	657	1.70 (0.62)	.684, *p* < .001 (1,508)	.671, *p* < .001 (634)	*z* = 0.51, *p* = .610
Mental health	1,508	1.62 (0.72)	634	1.48 (0.67)			
Secondary							
Victimization	771	1.75 (0.72)	405	1.63 (0.65)	.748, *p* < .001 (754)	.668, *p* < .001 (395)	***z* = 2.59, *p* = .010**
Mental health	754	1.59 (0.79)	395	1.55(0.77)			

Sample sizes differ for each variable and correlation due to missing data; significant differences are bolded for ease of presentation. Significant differences that are underlined did not remain significant after the BH correction.

**Table 3:** Means, standard deviations, and bivariate correlations between forms of bullying victimization and mental health before and during the COVID-19 pandemic for the overall sample.

	Pre-Covid *n*	Pre-Covid *M(SD)*	Pandemic *n*	Pandemic *M(SD)*	Pre-Covid *r(n)*	Pandemic *r(n)*	*r*-to-*z* test
Overall							
General Vic	2,261	1.89 (1.10)	1,013	1.62 (0.93)	.604, *p* < .001 (2,214)	.508, *p* < .001 (992)	***z* = 3.64, *p* < .001**
Physical Vic	2,254	1.48 (0.81)	1,021	1.36 (0.73)	.369, *p* < .001 (2,206)	.309, *p* < .001 (1,001)	*z* = 1.78, *p* = .075
Verbal Vic	2,269	2.08 (1.14)	1,029	1.92 (1.04)	.577, *p* < .001 (2,221)	.507, *p* < .001 (1,008)	***z* = 2.61, *p* = .009**
Social Vic	2,272	2.17 (1.11)	1,021	1.96 (1.03)	.578, *p* < .001 (2,222)	.508, *p* < .001 (999)	***z* = 2.61, *p* = .009**
Cyber Vic	2,269	1.34 (0.77)	1,022	1.41 (0.79)	.394, *p* < .001 (2,220)	.359, *p* < .001 (1,003)	*z* = 1.07, *p* = .285
Mental health	2,262	1.61 (0.74)	1,029	1.51 (0.71)			

Vic = bullying victimization; sample sizes differ for each variable and correlation due to missing data; significant differences are bolded for ease of presentation.

**Table 4:** Means, standard deviations, and bivariate correlations between forms of bullying victimization and mental health before and during the COVID-19 pandemic by gender.

	Pre-Covid *n*	Pre-Covid *M(SD)*	Pandemic *n*	Pandemic *M(SD)*	Pre-Covid *r(n)*	Pandemic *r(n)*	*r*-to-*z* test
Girls							
General Vic	1,198	1.86 (1.08)	473	1.61 (0.93)	.607, *p* < .001 (1,168)	.521, *p* < .001 (463)	***z* = 2.30, *p* = .021**
Physical Vic	1,191	1.35 (0.73)	480	1.23 (0.54)	.349, *p* < .001 (1,159)	.280, *p* < .001 (472)	*z* = 1.40, *p* = .162
Verbal Vic	1,205	2.04 (1.13)	485	1.87 (0.98)	.598, *p* < .001 (1,173)	.504, *p* < .001 (475)	***z* = 2.48, *p* = .013**
Social Vic	1,208	2.25 (1.09)	485	2.05 (0.97)	.569, *p* < .001 (1,176)	.422, *p* < .001 (475)	***z* = 3.59, *p* < .001**
Cyber Vic	1,196	1.38 (0.82)	478	1.42 (0.79)	.422, *p* < .001 (1,164)	.397, *p* < .001 (469)	*z* = 0.55, *p* = .582
Mental health	1,193	1.65 (0.76)	485	1.54 (0.66)			
Boys							
General Vic	922	1.88 (1.09)	470	1.60 (0.92)	.603, *p* < .001 (908)	.486, *p* < .001 (461)	***z* = 2.91, *p* = .004**
Physical Vic	919	1.63 (0.86)	468	1.48 (0.87)	.421, *p* < .001 (906)	.431, *p* < .001 (459)	*z* = −0.21, *p* = .834
Verbal Vic	920	2.10 (1.15)	470	1.90 (1.03)	.557, *p* < .001 (907)	.444, *p* < .001 (461)	***z* = 2.64, *p* = .008**
Social Vic	919	1.99 (1.07)	465	1.81 (1.03)	.563, *p* < .001 (904)	.533, *p* < .001 (456)	*z* = 0.74, *p* = .459
Cyber Vic	928	1.27 (0.67)	471	1.35 (0.74)	.306, *p* < .001 (914)	.278, *p* < .001 (463)	*z* = 0.54, *p* = .589
Mental health	923	1.50 (0.65)	471	1.40(0.65)			

Vic = bullying victimization; sample sizes differ for each variable and correlation due to missing data; significant differences are bolded for ease of presentation.

**Table 5:** Means, standard deviations, and bivariate correlations between forms of bullying victimization and mental health before and during the COVID-19 pandemic by grade level.

	Pre-Covid *n*	Pre-Covid *M(SD)*	Pandemic *n*	Pandemic *M(SD)*	Pre-Covid *r(n)*	Pandemic *r(n)*	*r*-to-*z* test
Elementary							
General Vic	1,500	1.96 (1.14)	620	1.68 (0.99)	.589, *p* < .001 (1,470)	.544, *p* < .001 (609)	*z* = 1.37, *p* = .171
Physical Vic	1,493	1.60 (0.88)	622	1.49 (0.83)	.373, *p* < .001 (1,462)	.330, *p* < .001 (612)	z = 1.02, *p* = .308
Verbal Vic	1,506	2.08 (1.16)	633	1.98 (1.07)	.577, *p* < .001 (1,475)	.492, *p* < .001 (622)	***z* = 2.49, *p* = .013**
Social Vic	1,510	2.19 (1.12)	622	1.94 (1.04)	.542, *p* < .001 (1,477)	.493, *p* < .001 (610)	*z* = 1.39, *p* = .165
Cyber Vic	1,509	1.29 (0.74)	621	1.32 (0.71)	.327, *p* < .001 (1,477)	.275, *p* < .001 (612)	*z* = 1.19, *p* = .234
Mental health	1,508	1.62 (0.72)	634	1.48 (0.67)			
Secondary							
General Vic	761	1.77 (1.00)	393	1.54 (0.82)	.646, *p* < .001 (744)	.474, *p* < .001 (383)	***z* = 4.01, *p* < .001**
Physical Vic	761	1.25 (0.60)	399	1.17 (0.48)	.407, *p* < .001 (744)	.383, *p* < .001 (389)	*z* = 0.45, *p* = .653
Verbal Vic	763	2.09 (1.11)	396	1.85 (0.99)	.621, *p* < .001 (746)	.547, *p* < .001 (386)	*z* = 1.79, *p* = .073
Social Vic	762	2.13 (1.10)	399	1.99 (1.01)	.647, *p* < .001 (745)	.533, *p* < .001 (389)	***z* = 2.80, *p* = .005**
Cyber Vic	760	1.45 (0.84)	401	1.54 (0.87)	.511, *p* < .001 (743)	.445, *p* < .001 (391)	*z* = 1.37, *p* = .171
Mental health	754	1.59 (0.79)	395	1.55(0.77)			

Vic = bullying victimization; sample sizes differ for each variable and correlation due to missing data; significant differences are bolded for ease of presentation.

For overall bullying victimization, there were significant reductions in correlations between bullying victimization and mental health difficulties for: (a) the overall sample, (b) girls, and (c) secondary students. There were also significant reductions in the correlations between some forms of bullying victimization and mental health difficulties. For the overall sample and girls, there were significant reductions in correlations with mental health difficulties for general, verbal, and relational bullying victimization. For boys, reductions in correlations were seen for general and verbal bullying victimization, whereas for secondary students there were reductions in correlations for general and relational victimization. Finally for elementary students, there was a reduction in the correlation for verbal bullying victimization. After applying the BH correction for multiple testing, the *r*-to-*z* test for the overall sample for overall bullying victimization was no longer significant and all other comparisons remained significant. In general, the most common forms of bullying victimization that saw significant decreases in correlations with mental health difficulties were for general, verbal, and relational, but not physical or cyber bullying victimization.

Results of the independent samples *t*-test revealed significant decreases in most forms of bullying victimization and mental health for the overall sample, for girls, boys, elementary students, and secondary students with two exceptions (see Table 6). The first exception was that there were no significant differences in cyber bullying victimization for all groups, except for the overall sample where there was significantly higher cyber victimization during the pandemic compared to before the pandemic.^2^ The second exception was that there was no significant difference in mental health difficulties for secondary students during the pandemic compared to before the pandemic. After applying the BH correction, the mean differences that were no longer significant were for cyber bullying victimization for boys, social bullying victimization for secondary students, and verbal bullying victimization for elementary students. All other differences remained significant. All mean differences were small in effect size.

**Table 6:** Independent *t*-tests comparing mean forms of bullying victimization and mental health before and during the COVID-19 pandemic.

	*df*	*t*-test	*p*	Cohen's *d*
Overall				
Victimization	2,324.45	−5.33***	**<.001**	−0.19
General Vic	3,272	−6.78***	**<.001**	−0.33
Physical Vic	2,174.90	−4.27***	**<.001**	−0.15
Verbal Vic	2,174.17	−3.87***	**<.001**	−0.14
Social Vic	2,106.41	−5.25***	**<.001**	−0.19
Cyber Vic	1,942.33	2.21*	**.027**	0.08
Mental health	2,068.31	−3.61***	**<.001**	−0.13
Gender				
Girls				
Victimization	1,113.22	−3.91***	**<.001**	−0.19
General Vic	1,169	−4.37***	**<.001**	−0.24
Physical Vic	1,178.64	−3.47***	**<.001**	−0.17
Verbal Vic	1,688	−2.90**	**.004**	−0.16
Social Vic	995.34	−3.69***	**<.001**	−0.19
Cyber Vic	1,672	0.98	.326	0.05
Mental health	1,021.61	−2.96**	**.003**	−0.15
Boys				
Victimization	1,051.17	−3.61***	**<.001**	−0.20
General Vic	1,390	−4.86***	**<.001**	−0.28
Physical Vic	1,385	−3.03**	**.002**	−0.17
Verbal Vic	1,388	−3.16**	**.002**	−0.18
Social Vic	1,382	−2.93**	**.003**	−0.17
Cyber Vic	869.00	2.15*	**.032**	0.12
Mental health	1,392	−2.78**	**.006**	−0.16
Grade				
Elementary				
Victimization	1,420.991	−4.35***	**<.001**	−0.19
General Vic	2,118	−5.28***	**<.001**	−0.25
Physical Vic	2,113	−2.73**	**.006**	−0.13
Verbal Vic	1,285.356	−2.08*	**.038**	−0.10
Social Vic	1,236.65	−4.92***	**<.001**	−0.23
Cyber Vic	2,128	0.97	.331	0.05
Mental health	1,266.04	−4.17***	**<.001**	−0.19
Secondary				
Victimization	899.93	−2.84**	**.005**	−0.17
General Vic	935.12	−4.21***	**<.001**	−0.25
Physical Vic	981.11	−2.76**	**.006**	−0.16
Verbal Vic	1,157	−3.52***	**<.001**	−0.22
Social Vic	865.38	−2.13*	**.033**	−0.13
Cyber Vic	1,159	1.72	.086	0.11
Mental health	1,147	−0.71	.479	−0.04

Condition coded as 0 = Pandemic and 1 = Pre-Covid; Vic, victimization. Significant differences are bolded for ease of presentation. Significant differences that are underlined did not remain significant after the BH correction.

* *p* < .05. **p < .01. ***p < .001.

## 4 Discussion

We examined whether the association between bullying victimization and mental health difficulties specifically due to victimization was different from before to during the COVID-19 pandemic using a population-based randomized design. Several studies from early in the pandemic revealed significant declines in prevalence rates of bullying victimization (20, 21). Therefore, we examined whether the association between victimization and mental health difficulties specifically due to bullying would also decrease after the pandemic onset. Our predictions were largely supported as the correlations between bullying victimization and mental health were significant before and during the pandemic but were significantly weaker during the pandemic. This pattern was found for overall bullying victimization among girls (but not boys) and secondary students (but not elementary students). When broken down by form of bullying victimization, the most common forms of victimization that reflected this pattern were general, verbal, and social victimization. Patterns for all three forms were found for the overall sample and girls. For boys, this pattern was found for general and verbal victimization, whereas for secondary students, this pattern was found for general and social victimization. For elementary students, this pattern was found for verbal victimization only.

The consistent pattern of a decline in the correlation between bullying victimization and mental health due to victimization is likely due to the significant decline in prevalence rates early in the pandemic. Several studies revealed significant correlations between bullying victimization and indicators of mental health difficulties before and during the COVID-19 pandemic, with correlations during the pandemic appearing smaller than before the pandemic (21, 31). However, the magnitude of the correlations before compared to during the pandemic were not statistically compared in these studies. In our study, correlations between bullying victimization and mental health difficulties due to victimization were also significant before and during the pandemic, although we found statistically significant decreases in correlation magnitudes during the pandemic. In addition, we found that mean levels of bullying victimization decreased for most forms, except cyber bullying victimization. This suggests that the intensity and/or the frequency of victimization could have decreased during the pandemic. We also found that mean levels of mental health difficulties specifically due to victimization decreased overall and for all groups except for secondary school students. It is important to note that the effect size in these previous studies were small-to-moderate before the pandemic (*r* = .21–.30) and small during the pandemic (*r* = .09–.15). In contrast, in our study the effect sizes ranged from small-to-moderate to moderate-to-large before (*r* = .31–.75) and during the pandemic (*r* = .28–.67). Our effect sizes are likely larger than previous studies because we asked students about their mental health experiences specifically related to being victimized among students who reported being victimized, whereas previous studies asked students about mental health experiences in general.

We also found decreases in correlation magnitude for overall bullying victimization for girls and for secondary school students, and predominately for general, social, and verbal forms of bullying victimization. The trends by demographic factors and victimization forms appear to support some common patterns of bullying. In Canada, girls are often targeted in bullying more than boys overall and by most forms (9, 10). The social restrictions put in place due to the pandemic may have provided an escape for girls from the higher frequency of victimization and subsequently mental health difficulties, at least relative to boys. In addition, bullying prevalence rates are lower in secondary students compared to elementary students (11, 34). The significant drop in correlation among secondary students but not elementary students may indicate that pandemic mitigation measures were more effective in reducing rates of bullying victimization in older students. It also appeared that social bullying victimization was significantly lower during the pandemic for elementary students, but verbal bullying victimization was significantly lower for secondary students.

These differences in correlations could be driven by the decreases in victimization rather than mental health difficulties. Among victimized students, secondary students were the only group without significant differences in mental health difficulties due to victimization during the pandemic compared to before the pandemic, despite significantly lower victimization rates during the pandemic. Indeed, researchers have previously documented the enduring mental health consequences of being bullied (5). Therefore, the nonsignificant difference in secondary students' mental health during the pandemic could reflect the persistent impacts of being victimized, whereas these lasting effects may not yet be entrenched in younger elementary students.

We found largely consistent patterns in reduced correlation magnitudes for general, social, and verbal bullying victimization. These forms of bullying victimization are often more commonly seen in older elementary and secondary school students, relative to physical bullying which is more commonly seen in younger elementary students (10). Therefore, pandemic related mitigation practices may have been more effective in reducing these developmentally salient forms of bullying victimization. It is curious that the correlation between physical victimization and mental health difficulties due to victimization did not decrease, given that social distancing practices should have decreased physical contact. However, examining the mean levels of the forms revealed that physical victimization had lower mean levels before and during the pandemic relative to the other forms of victimization, despite mean levels of physical victimization being significantly lower during the pandemic compared to pre-pandemic for all groups. We also did not find reductions in the correlation between cyber victimization and mental health due to victimization. Indeed, we found no significant differences in cyber victimization before and during the pandemic, and instead found that cyber victimization was higher during the pandemic than before the pandemic for the overall sample among individuals who are victimized. Thus, among individuals who were victimized, cyber victimization may have been a common form experienced during the pandemic relative to other forms of bullying. These mean levels combined with the likelihood that cyber victimization can be experienced outside the physical confounds of a school setting may be why we did not find significant reductions between cyber victimization and mental health difficulties due to victimization.

Our findings highlight the strong coupling of bullying victimization and mental health difficulties due to victimization, especially before the pandemic. These findings also indicate the need to differentiate the mechanisms between adverse mental health due to being victimized and adverse mental health more broadly. For example, it is evident through several meta-analyses that mental health difficulties increased for many children and adolescents during the pandemic, with more pronounced effects in girls and older adolescents [e.g., (15)]. However, our results reflect a weaker association between bullying victimization and mental health specifically due to being victimized. We found that mean levels of bullying victimization and mental health difficulties were lower during the pandemic than before the pandemic among students who are bullied. The two exceptions were for cyber bullying victimization, which was higher for the overall sample during the pandemic and did not change for the other groups, and for secondary students who had no difference in mental health difficulties during the pandemic. It is likely that there are heterogeneous developmental pathways contributing to mental health difficulties in children and adolescents (35, 36). For some individuals, pandemic mitigation measures including social distancing, increased educator supervision, and smaller classroom sizes unintentionally resulted in decreased bullying victimization and provided a reprieve from the stressors of being victimized. For other students, including students who are not victimized, these pandemic measures could have contributed to increased feelings of social isolation and low social support, that could have contributed to or exacerbated mental health difficulties. Indeed, evidence suggests increases in loneliness among children and adolescents during the pandemic (37). Heterogeneous developmental pathways should continue to be examined to improve child and adolescent mental health.

### 4.1 Limitations

Despite the contributions of our study, there are some limitations. First, students in the pre-pandemic condition responded to a 6-month timeframe whereas students in the pandemic condition responded to a 3-month timeframe. These differences could have inflated our results, but as noted by Vaillancourt et al. (20), the pre-pandemic prevalence rates of these data map onto prevalence rates of pre-pandemic studies using a 3-month timeframe (10). In addition, the pre-pandemic assessments relied on memory from a more distant time frame, but literature on social pain indicates that victimization is such a salient experience that it can be accurately recalled (38). Second, we examined associations only among students who were bullied because the mental health items were specific to experiences of being bullied. Researchers should examine whether reductions in correlations are replicated between bullying victimization and overall mental health (i.e., not specific to bullying) or if different patterns emerge. The use of general measures of mental health difficulties along with measures of mental health difficulties due to victimization in future studies could also reveal nuances between victimization and mental health. Third, we attributed the reduced magnitude of correlations between bullying victimization and mental health to decreases in bullying prevalence rates early in the pandemic. Although the randomized population-based design is a strength of this study, our data were measured concurrently, and thus longitudinal data are needed to test this theorized direction of effects. For example, existing studies with data from before the pandemic could allow for controlling for pre-pandemic mental health difficulties to see how mental health was impacted by bullying victimization after the pandemic onset. In addition, the association between bullying perpetration and overall mental health before and after the pandemic onset could be examined. Fourth, it is likely that students are victimized in multiple forms. Although we examined each form of victimization separately, person-centered approaches in future studies can reveal whether the forms of victimization experienced by the same students changed before the pandemic compared to during the pandemic. Fifth, we used self-report measures, which could contribute to shared method variance and social desirability bias. The use of additional reporters (e.g., peer nominations for bullying victimization) could be included in future studies.

### 4.2 Conclusions and implications

Our study revealed that the well-established correlation between bullying victimization and mental health was significantly smaller during the pandemic compared to before the pandemic. Reducing bullying victimization can therefore significantly reduce associated mental health difficulties due to victimization. It is also important to recognize that all correlations between bullying victimization and mental health difficulties during the pandemic were statistically significant, despite significant reductions. Bullying and mental health difficulties remain a problem, but as the pandemic data suggests, it is possible to reduce the prevalence of victimization. Some of the pandemic mitigation practices may have unintentionally reduced bullying, such as more caring teacher supervision and monitoring [e.g., (20)]. The pandemic provided a “natural experiment” for the effectiveness of these features. Continuing to focus on and implement supportive and supervised classrooms on a larger scale can foster long-term positive school climates, reduce bullying, and hopefully any subsequent mental health difficulties. This idea is consistent with meta-analytic results which have revealed that the most effective bullying interventions include a whole school approach that includes the involvement of school administrators, educators, staff, and students (39). By increasing supportive and caring social relationships, we can reduce bullying, improve mental health, and contribute to the healthy development of children and adolescents.

## Data Availability

The raw data supporting the conclusions of this article will be made available by the authors, without undue reservation.

## Footnotes

^1^Due to the small number of participants identifying as gender diverse, we only examined girls and boys for subsequent analyses involving comparisons by condition.

^2^The higher mean levels of cyber bullying victimization for the overall sample during the pandemic compared to before the pandemic was only among students *who reported being victimized*. Note that Vaillancourt et al. (20) used the same dataset and found significantly lower prevalence rates of victimization for all forms of bullying, including cyber bullying, during the pandemic compared to before the pandemic. However, they specifically compared frequencies of *no involvement* versus *any involvement* in victimization.
